# Multi-region saliency-aware learning for cross-domain placenta image segmentation

**DOI:** 10.1016/j.patrec.2020.10.004

**Published:** 2020-12

**Authors:** Zhuomin Zhang, Dolzodmaa Davaasuren, Chenyan Wu, Jeffery A. Goldstein, Alison D. Gernand, James Z. Wang

**Affiliations:** a The Pennsylvania State University, University Park, PA, USA; b Northwestern University, Chicago, IL, USA

**Keywords:** Transfer learning, Placenta, Photo image analysis, Pathology

## Abstract

We propose a multi-region saliency-aware learning (MSL) method for cross-domain placenta image segmentation. Unlike most existing image-level transfer learning methods that fail to preserve the semantics of paired regions, our MSL incorporates the attention mechanism and a saliency constraint into the adversarial translation process, which can realize multi-region mappings in the semantic level. Specifically, the built-in attention module serves to detect the most discriminative semantic regions that the generator should focus on. Then we use the attention consistency as another guidance for retaining semantics after translation. Furthermore, we exploit the specially designed saliency-consistent constraint to enforce the semantic consistency by requiring the saliency regions unchanged. We conduct experiments using two real-world placenta datasets we have collected. We examine the efficacy of this approach in (1) segmentation and (2) prediction of the placental diagnoses of fetal and maternal inflammatory response (FIR, MIR). Experimental results show the superiority of the proposed approach over the state of the art.

## Highlights

• Established a cross-domain dataset consisting of placenta photos and medical records.
• Designed attention and saliency-guided constraints to realize multi-object translation.
• Devised an integrated pipeline for cross-domain placenta image segmentation.
• Showed potential of our MSL model in detecting significant pathological/abnormal indicators.

## 1 Introduction

**Fig. 1:**
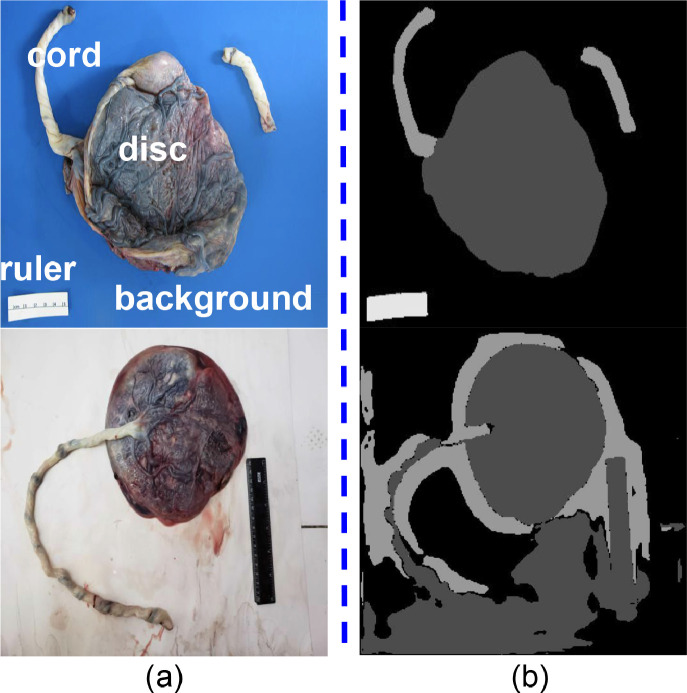
Challenges for cross-domain placenta image segmentation. (a) Cross-domain images: Placenta images from two different hospitals. (b) Their corresponding segmentation results using the trained model on the first dataset.

The placenta is the essential connection between mother and fetus, sensing nutrient availability and needs, producing hormones to drive physiologic changes, and protecting the fetus from pathogens [18]. Because the main function of the placenta is to support important metabolic activities, pathological analysis of the placenta should be an integral part of the health examination during pregnancy and after delivery. Yet, as a result of costly examination charges and limited expertise and facilities in developing countries, we estimate that only a small proportion of placentas ever examined by a pathologist worldwide. Automating pathological analysis by advanced image processing techniques is an inevitable trend because it can substantially augment the productivity of pathologists, shorten examination time and enhance examination accuracy. Particularly, accurate image segmentation is essential for integrated computerized placenta photo analysis [2], [3]. As shown in Fig. 1, the placenta disc, umbilical cord, ruler (for measuring the scale), and background are four common categories that must be segmented in a photo for further analysis.

Although convolutional neural networks (CNNs) have triumphed over conventional object segmentation in many clinical image segmentation and analysis applications [2], [3], [10], [16], the generalizability of a trained CNN is often inadequate when applied to a new dataset (e.g., photos from a different hospital) because the two datasets often have vastly different data distributions [24], [25].

This limits the applicability of AI models trained on data from high-resource settings, such as academic medical centers, to lower resource settings including community hospitals and low income countries. As illustrated in Fig. 1(a), although the appearances of the disc and cord are reasonably consistent across different data sources, the ruler and background can be vastly distinctive in terms of the color, texture, and amount of distraction. These largely different visual appearances can cause a well-trained CNN model, such as UNet [20], to be vulnerable when generating segmentation results in an unseen domain, as shown in Fig. 1(b). Because of the difficulties lying in data collection and annotation, it is practically infeasible to retrain a label-dependent CNN model to adapt to different datasets every time.

Therefore, it is highly desirable to close the gap across domains by an effective domain adaptation method. This paper focuses on the domain adaptation problem for placenta image segmentation when data in the target domain are insufficient and the corresponding labels are unavailable.

Most existing unsupervised domain adaptation methods can be categorized into: (1) feature distribution alignment, (2) task-specific output space relation preservation, and (3) image-to-image translation. Feature-level adaptation methods aim at aligning the two domains in a latent feature space by minimizing the distribution distance, such as the maximum mean discrepancy [13], or leveraging adversarial learning strategies [6]. However, the aligned feature space is not guaranteed to be semantically consistent and some low-level visual cues that are crucial for the end segmentation task may be lost. The output space adaptation method aims to make the predicted maps close to each other [4], [22]. It is not suitable for placenta image segmentation because the spatial relations among objects are not consistent across domains. Image-to-image translation tries to alleviate the domain shift from headstream by forcing the cross-domain images to look like those from the original domain. Some regularization terms such as the cycle strategy [26] are exploited to make the training process free of paired data. Attention mechanism is also introduced to pair the regions of interests across domains for more realistic image generation [1], [15]. Nevertheless, these attention methods only focus on one specific type of object, so the translation can still be mismatched when multiple objects are attended to simultaneously. How to enforce semantic consistency for multi-region translation is the research problem our MSL model aims to solve.

In this paper, we propose a multi-region saliency-aware learning method to realize cross-domain placenta image translation by enforcing both the attention and saliency consistency. An attention module serving as the semantic guidance is firstly coupled with the classic generator-discriminator game to find the most discriminative regions (i.e., ruler and background). Out of the motivation for semantic-consistent transfer of multi regions, an attention-consistent loss is added as an extra constraint to enforce the translation to preserve the attention-related information. Notably, we devise a new saliency-consistent constraint as another semantic guidance by restraining the saliency relation unchanged after translation. Finally, we feed the translated target domain images to a well-trained CNN model from the annotation-sufficient source domain for the ultimate segmentation task.

## 2 Approach

**Fig. 2:**
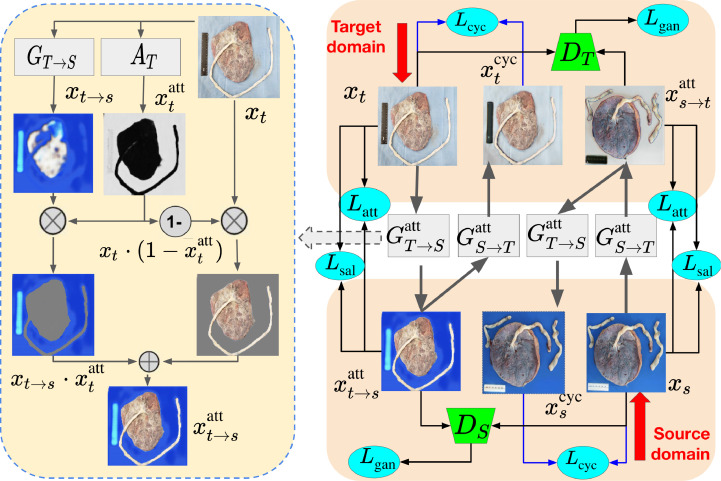
The pipeline of the proposed approach. As shown in the left part, the introduced attention network *A_T_* can divide the placenta image *x_t_* into attended regions such as ruler and background, and unattended regions that include disc and cord. The translated image $x_{t\to s}^{\text{att}}$ is a combination of translated attended parts and original unattended parts. The attention-consistent loss $L_{\text{att}}$ and a saliency-consistent $L_{\text{sal}}$ loss are added to preserve the semantics in together with the image-level adaptation as composed of the pixel GAN loss *L*_gan_ and the cycle loss *L*_cyc_.

The overall framework of our MSL is presented in Fig. 2, where two attention networks, *A_S_* and *A_T_*, work together with corresponding generators *G*_*S* → *T*_ and *G*_*T* → *S*_, along with two discriminators *D_S_* and *D_T_*, to form our MSL model. Due to the high demand of multi-region semantic consistency before and after image translation for our end segmentation task, we add an attention-consistent loss $L_{\text{att}}$ to alleviate the influence of unattended regions and a simple but effective saliency-consistent constraint $L_{\text{sal}}$ to guarantee the salient regions to stay unaltered during the generation. Meanwhile, the classic adversarial loss *L*_gan_ and cycle loss *L*_cyc_ are exploited together to accomplish semantic mappings, which will be described in detail as follows.

We formulate this task as an image-to-image translation for segmentation map prediction. We assume that the source image set *X_S_* together with the source label set *Y_S_* are accessed, while only the image set *X_T_* in the target domain is available. Our final goal is to adapt the pre-trained segmentation model *F_S_* to the translated source-like images in the target domain.

### 2.1 Cycle generative adversarial network

The goal of image translation is to learn a mapping between the source domain and the target domain. A generative model annotated as *G*_*S* → *T*_ is exploited to learn this kind of data mapping to generate target-like images $x_{s\to t}=G_{S\to T}\left(x_{s}\right),$ which can deceive the discriminator. On the contrary, the discriminator *D_T_* aims to distinguish the genuine image *x_t_* from the translated images *x*_*s* → *t*_, to constitute a dynamic min-max two-player game. We adopt the adversarial loss function in LSGAN [14] into our model, and *L*_gan_ is denoted as:

(1) $$\begin{array}{ccc} L_{\text{gan}}\left(G_{S\to T},D_{T},X_{S},X_{T}\right) & = & E_{x_{t}∼P_{X_{T}}\left(x_{t}\right)}\left[\log\left(D_{T}\left(x_{t}\right)\right)\right]\\ & & +\;E_{x_{s}∼P_{X_{S}}\left(x_{s}\right)}\left[\log\left(1-D_{T}\left(x_{s\to t}\right)\right)\right]\;. \end{array}$$

Similarly, the corresponding loss function for the target-to-source translation *L*_gan_(*G*_*T* → *S*_, *D_S_, X_T_, X_S_*) is defined in the same way. That is,

(2) $$\begin{array}{ccc} L_{\text{gan}}\left(G_{T\to S},D_{S},X_{T},X_{S}\right) & = & E_{x_{s}∼P_{X_{S}}\left(x_{s}\right)}\left[\log\left(D_{S}\left(x_{s}\right)\right)\right]\\ & & +\;E_{x_{t}∼P_{X_{T}}\left(x_{t}\right)}\left[\log\left(1-D_{S}\left(x_{s\to t}\right)\right)\right]\;. \end{array}$$

The discriminators *D_S_* and *D_T_* attempt to maximize the loss, while the generators *G*_*S* → *T*_ and *G*_*T* → *S*_ strive to minimize the loss. To make the translated image preserve the structure and local content of the original image, a cycle consistency loss [8] is also designed as follows by measuring the pixel-wise difference between the reconstructed image and the original image.

(3) $$\begin{array}{ccc} L_{\text{cyc}}\left(G_{T\to S},G_{S\to T}\right) & = & E_{x_{s}∼P_{X_{S}}\left(x_{s}\right)}[∥G_{T\to S}\left(x_{s\to t}\right)-x_{s}{∥}_{1}]\\ & & +\;E_{x_{t}∼P_{X_{T}}\left(x_{t}\right)}[∥G_{S\to T}\left(x_{t\to s}\right)-x_{t}{∥}_{1}]\;. \end{array}$$

### 2.2 Multi-region saliency-aware constraints

To realize multi-region translation simultaneously by a single generator, we made the assumption that fixing the foreground regions (placenta disc and cord) would improve the translation quality and subsequent cross-domain pathological diagnosis adaptation. The rationales are: (1) The characteristics of placenta datasets: Although the background material and the ruler of placenta photos vary a lot across different clinical datasets, visual differences of the foreground disc and cord are relatively negligible because placentas share the same basic structure and appearance. We have collected over 1000 placentas in the source dataset covering most variations in placenta morphology in the target domain, so the feature extracted from the placenta is insignificant for fake image discrimination. That also explains why the foreground is learned as unattended regions. Hence, transferring the foreground placenta itself helps little to the quality of the final generated images. (2) The requirement of real clinical application: The pathological indicator prediction relies on the extracted visual features from the placenta, so keeping the original foreground features unchanged is essential for adapting the pretrained source-domain diagnosis models to the translated images. Otherwise, the diagnosis results could be unreliable if the foreground is also translated. (3) The functionality of the generator: Incorporating the separation and conversion of foreground objects with the existing background translation in a single network would confuse the aims of the generator, leading to unmatched contents in generated images. This viewpoint has been justified in [1,13], where fixing the unattended regions can help generate more realistic images than transferring the entire image.

Inspired by AGGAN [15], we decompose the generative module into two separated parts: (1) the attention networks *A_S_* and *A_T_* attempting to attend to the regions that the discriminator considers to be the most descriptive within its domain (i.e., the ruler and background in our application), and (2) the classic generative networks focusing on transforming the whole image from one domain to another. The ultimate generated image is therefore a combination of the attended regions from the transformed image and unattended areas in the original image by using the attention map as mask. With the attention mechanism, the discriminator can force the attention networks to find the most domain-descriptive regions of interest and, therefore, make the generators pay more attention to the attended objects.

We denote the attention maps induced from *X_S_* and *X_T_* as $x_{s}^{\text{att}}=A_{S}\left(x_{s}\right)$ and $x_{t}^{\text{att}}=A_{T}\left(x_{t}\right),$ respectively. Attention-guided generated map can then be computed as:

(4) $$x_{s\to t}^{\text{att}}=x_{s}^{\text{att}}\cdot G_{S\to T}\left(x_{s}\right)+\left(1-x_{s}^{\text{att}}\right)\cdot x_{s}\;,$$

where  ·  denotes the element-wise product. This attention network is jointly adversarially trained with the generators and discriminators. We replace *x*_*s* → *t*_ and *x*_*t* → *s*_ in the (1) and (3) with $x_{s\to t}^{\text{att}}$ and $x_{t\to s}^{\text{att}},$ respectively.

*Attention-consistent loss* One regulation of generators is that the transformed image should have the same semantics as the original image. If the semantics are aligned, in other words, the attended regions should maintain the same before and after translation: *A_S_*(*x_s_*) ≈ *A_T_*(*x*_*s* → *t*_). Instead of using the segmentation label to supervisedly preserve the semantics [12], we treat the learned attention map as an important form of semantics. Besides, because we have the segmentation maps in the source domain, we can add extra supervision to attention map generation in the source domain. To that end, the attention-consistent losses are formulated as:

(5) $$\begin{array}{ccc} L_{\text{att}}\left(A_{S}\right) & = & E_{x_{s}∼P_{X_{S}}\left(x_{s}\right)}[∥A_{S}\left(x_{s}\right)-y_{s}{∥}_{1}]\\ & & +\;E_{x_{s}∼P_{X_{S}}\left(x_{s}\right)}[∥A_{T}\left(x_{s\to t}\right)-y_{s}{∥}_{1}]\;,\\ L_{\text{att}}\left(A_{T}\right) & = & E_{x_{t}∼P_{X_{T}}\left(x_{t}\right)}[∥A_{T}\left(x_{t}\right)-A_{S}\left(x_{t\to s}\right){∥}_{1}]\;. \end{array}$$

*Saliency-consistent loss* In most attention-guided image translation cases, the most salient object in the foreground is likely to be learned as the region of interest. To add the additional saliency-consistent loss is hence meaningless. However, this attention map is learned by the discriminator, which is not always consistent with the visual attention (i.e., saliency). For instance, what if both the background and foreground objects are included into the attended region? In our case, it is observed that the cord and disc in the foreground stay changeless across domains, while the ruler and background suffer a lot of variations, leading them to be classified as attended areas by the discriminator. Even if we force the generator to focus on this region, the generated ruler and background can still be mismatched. Therefore, adding the saliency-consistent loss as a constraint is indispensable to help maintain the semantic consistency before and after translation to prevent label flipping.

Because the attended areas can be obtained at the early training stage, we only compute the saliency value for pixels in the attended regions. We employ the simple but effective FT [17] method for saliency detection:

(6) $$S\left(i,j\right)=∥I\left(i,j\right)-I_{\mu }{∥}_{2}\;,$$

where *I*(*i, j*) represents the pixel color vector value after Gaussian blurring [7], and *I_μ_* is the mean image color vector. || · ||_2_ is the Euclidean distance between color vectors. We denote the saliency maps for both the images in the original domain and the translated images as *S_S_, S_T_* and *S*_*S* → *T*_, *S*_*T* → *S*_, respectively. We binarize these saliency maps *S_S_, S_T_* as the saliency ground truth to formulate the loss function. To overcome the problem that the number of pixels in different categories are highly unbalanced, the saliency consistency loss is defined using the dice coefficient [21]:

(7) $$L_{\text{sal}}\left(G_{S\to T}\right)=1-\frac{\sum_{i,j}S_{S}\left(i,j\right)\cdot S_{S\to T}\left(i,j\right)}{\sum_{i,j}S_{S}\left(i,j\right)+\sum_{i,j}S_{S\to T}\left(i,j\right)}\;.$$

The *L*_sal_(*G*_*T* → *S*_) is defined in a similar way.

We obtain the final loss function by combining the adversarial, cycle consistency, attention consistency, and saliency consistency losses for both the source and target domains, defined as:

(8) $$\begin{array}{ccc} L_{\text{total}} & = & L_{\text{gan}}\left(G_{S\to T},D_{T},X_{S},X_{T}\right)+L_{\text{gan}}\left(G_{T\to S},D_{S},X_{T},X_{S}\right)\\ & & +\;\lambda_{\text{cyc}}L_{\text{cyc}}\left(G_{T\to S},G_{S\to T}\right)+\lambda_{\text{att,S}}L_{\text{att}}\left(A_{S}\right)+\lambda_{\text{att,T}}L_{\text{att}}\left(A_{T}\right)\\ & & +\;\lambda_{\text{sal,S}}L_{\text{sal}}\left(G_{S\to T}\right)+\lambda_{\text{sal,T}}L_{\text{sal}}\left(G_{T\to S}\right)\;. \end{array}$$

This ultimate model parameters can be obtained by solving the mini-max optimization problem:

(9) $$G_{S\to T}^{*},G_{T\to S}^{*},D_{S}^{*},D_{T}^{*},A_{S}^{*},A_{T}^{*}=\underset{G_{S\to T},G_{T\to S},A_{S},A_{T}}{\arg\min}\underset{D_{S},D_{T}}{\arg\max}L_{\text{total}}\;.$$

*Segmentation loss* We adopt the same structure of the segmentation module from PlacentaNet [2] to train a segmentation model in the source domain. We use *p*(*i, j, k*) to denote the prediction probability of the pixel (*i, j*) belonging to class *k* and *g*(*i, j, k*) to represent the corresponding ground truth. Sharing the same spirit of saliency consistency loss to balance labels, the dice loss for 4-class segmentation is defined as:

(10) $$L_{seg}=1-\frac{\sum_{i,j}\sum_{k=0}^{3}p\left(i,j,k\right)\cdot g\left(i,j,k\right)}{\sum_{i,j}\sum_{k=0}^{3}\left(p\left(i,j,k\right)+g\left(i,j,k\right)\right)}\;,$$

We apply this pretrained model to the translated target-domain images to obtain the final segmentation results.

## 3 Experiments

### 3.1 Datasets and experimental settings

We curated real-world post-delivery datasets, including a relatively clean image set together with comprehensive pathology reports (de-identified) from a large urban academic hospital,the Northwestern Memorial Hospital, as the source domain, and images of non-professional quality taken from a hospital in Mongolia (only images, no accompanying pathology reports) as the target domains. A web-based annotation tool was developed to: (1) discard images that don’t meet our image quality standard (disc and cord should be fresh and not occluded by irrelevant objects); and (2) get pixel-wise segmentation maps for the disc, cord, ruler and background annotated by trained labelers. The dataset collected as the source domain contained 1003 placenta images together with segmentation maps and extracted diagnoses from the pathology reports, while the target dataset has 76 images and corresponding annotated segmentation maps for evaluation purpose.

We divided the dataset in the source domain into training and testing sets with the ratio of 0.8: 0.2 for training the segmentation model. For the image translation task, 200 images from the source domain and 60 images from the target domain were used for training. We used cross-validation to demonstrate the translation performance and the segmentation result in the target domain.

*PlacentaNet* We adopted the same encoder-decoder structure from PlacentaNet [2], [3] to train a segmentation model in the source domain. We used the Adam optimizer [11] with a mini-batch size of 5 and a learning rate of 0.001 for training. The pixel-wise accuracy and mean IoU are 0.9693 and 0.9131 respectively for testing in the same domain.

*Translation network* Our training process can be separated into three steps: (1) We first trained the discriminators on full images for 20 epochs to help the attention module well trained with the guidance of the attention consistency loss; (2) Then, we make the discriminator to focus on the salient region (i.e., the ruler) within the next 5 epochs by multiplying the saliency map (threshold = 0.7) to the image, which can alleviate the unbalanced label distribution problem. (3) Finally, we multiply the binarized attention map (threshold = 0.2) to the generated images to make the discriminator only consider attended regions. The saliency loss is then added to guide the overall translation performance.

For all steps, the training images were rescaled to 512 × 512 pixels, following by random flipping for data augmentation. We used Adam with a batch size of 1 and a linearly decaying learning rate from 0.0002 for the training of all the three networks. As for the network structure, we used the residual attention module introduced in Wang et al. [23] as attention network, Resnet-9blocks [9] as the generator and PatchGAN [8] as the discriminator. We set hyper-parameters $\lambda_{\text{cyc}}=5,$ $\lambda_{\text{att,S}}=2,$ $\lambda_{\text{att,T}}=4,$ $\lambda_{\text{sal,S}}=1,$ and $\lambda_{\text{sal,T}}=1,$ respectively.

### 3.2 Results

To show the improvement on segmentation brought by the cross-domain adaptation, we first compare our model with the baseline scenario (i.e., segmentation without adaptation). Then two state-of-the-art image translation models, CycleGAN [26] and AGGAN [15], are compared to demonstrate the superiority of our MSL model. These two methods are pioneering in the GAN-based translation methods and most relevant to our placenta segmentation problem. The segmentation performance is evaluated using standard segmentation metrics, including pixel accuracy, mean accuracy, and mean IoU. The definition of those metrics are as follows: we use *P*_*i,j*_denote the number of pixels that are annotated as class *i* but predicted as class *j*. The total number of pixels that belong to class *i* in the ground truth are denoted as *G_i_*. Because there are 4 classes (ruler, disc, cord and background) in our case, *i, j* ∈ {0, 1, 2, 3}. The pixel accuracy, mean class accuracy, and mean IoU are then defined as follows:

• Pixel accuracy:
  $\frac{\sum_{i=0}^{3}P_{i,i}}{\sum_{i=0}^{3}G_{i}}\;.$
• Mean class accuracy:
  $\frac{1}{4}\sum_{i=0}^{3}\frac{p_{i,i}}{G_{i}}\;.$
• Mean IoU:
  $\frac{1}{4}\sum_{i=0}^{3}\frac{P_{i,i}}{G_{i}+\sum_{j\neq i}P_{i,j}}\;.$

**Fig. 3:**
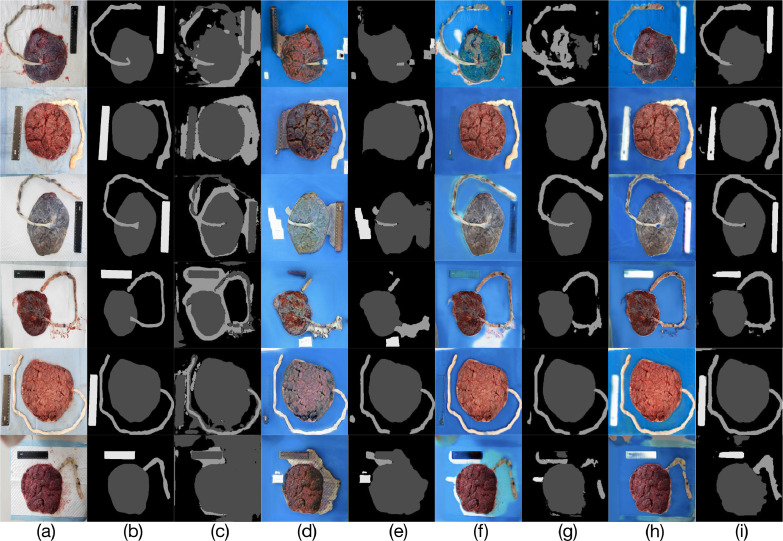
Segmentation result comparisons. (a) Original images. (b) Ground truth. (c) Segmentation results without adaptation. (d)(f)(h) Translation results using CycleGAN, AGGAN, and our model, respectively. (e)(g)(i) Segmentation results using the translated images to the left of it.

**Fig. 4:**
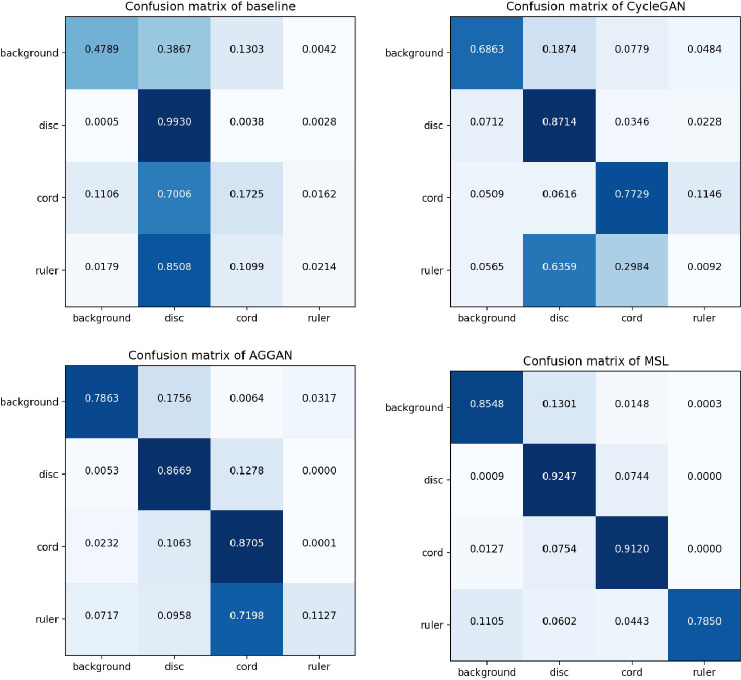
Confusion matrices of baseline, CycleGAN, AGGAN and MSL.

The quantitative results are shown in Table. 1. We observe that the segmentation performance has been greatly improved from 0.2049 to 0.7380 in mean IoU by adding proper adaptation. The success of the background translation leads to a big leap on the pixel accuracy. The improvement of Mean IoU should be credited to our accurate ruler translation with the saliency-aware guidance. We also show a few segmentation examples in Fig. 3 for qualitative comparison. Some special cases, including non-uniform background, poor-quality cloth, different color, and messy surrounding, are shown to demonstrate the robustness of our proposed translation method. Besides, to illustrate detailed segmentation performance in different categories,we also compare our approach with the baseline, AGGAN and CycleGAN respectively using pixel-wise prediction confusion matrices as shown in Fig. 4. According to the confusion matrices, it is noticeable that our method greatly improve the segmentation accuracy of cord and ruler with the added losses.

**Table 1:** Segmentation evaluation accuracy.

Method	Pixel Accu.	Mean Accu.	Mean IoU
No adaptation	0.5256	0.4164	0.2049
CycleGAN	0.7022	0.5850	0.3508
AGGAN	0.7807	0.6497	0.4672
MSL(w/o*L*_att_)	0.8291	0.6502	0.4812
MSL(w/o*L*_sal_)	0.7852	0.6591	0.4722
MSL(w/o*L*_cyc_)	0.7593	0.6203	0.3874
MSL	**0.8743**	**0.8691**	**0.7380**

### 3.3 Ablation study

To show the indispensability of each loss term, we conducted an ablation study as follows.

**Fig. 5:**
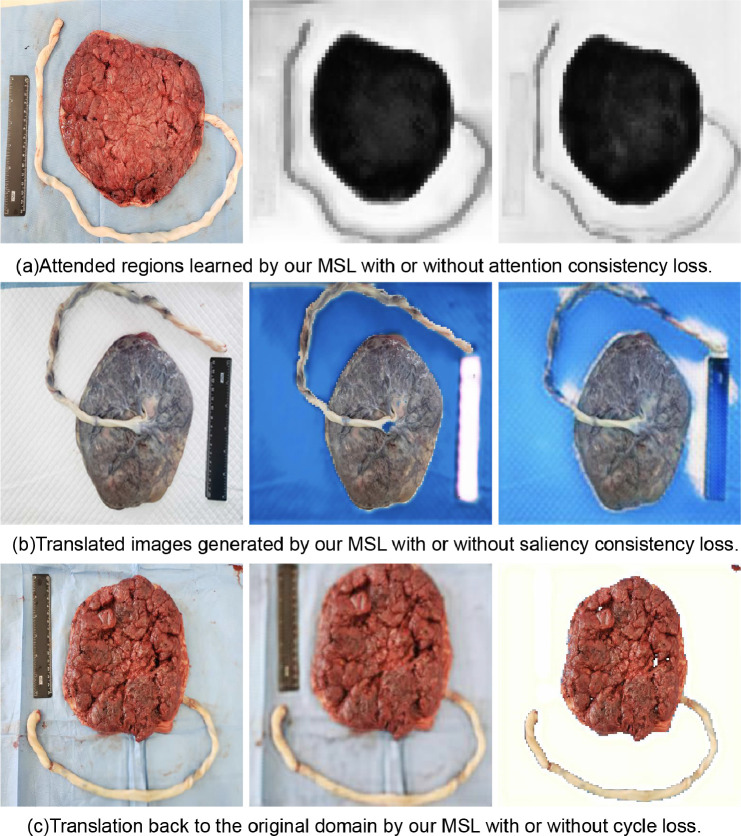
Ablation Study. Left: original images. Middle: results of MSL. Right: results without the corresponding loss.

*Effectiveness of attention consistency* As shown in the Fig. 5(a), the attention-consistent loss enforces the cord region to be unattended by keeping the consistency of attention regions. Without the attention-consistent loss, the cord can sometimes be learned as the attended background due to the similar light colors and bloody background in the target domain.

*Effectiveness of saliency consistency* Fig. 5(b) shows the saliency loss constrains the saliency relation between ruler and background to be consistent. Without the saliency constraint, the translation often suffers from random label flipping because the generator fails to produce matched semantics.

*Effectiveness of cycle consistency* From Fig. 5(c), we observe that the translation back to the original domain sometimes fails because there is no reconstruction guarantee without the cycle loss, which may cause the structure of the generated image altered. The quantitative results of removing each loss term can be found in the Table. 1.

### 3.4 Enhancing diagnosis of chorioamnionitis

**Fig. 6:**
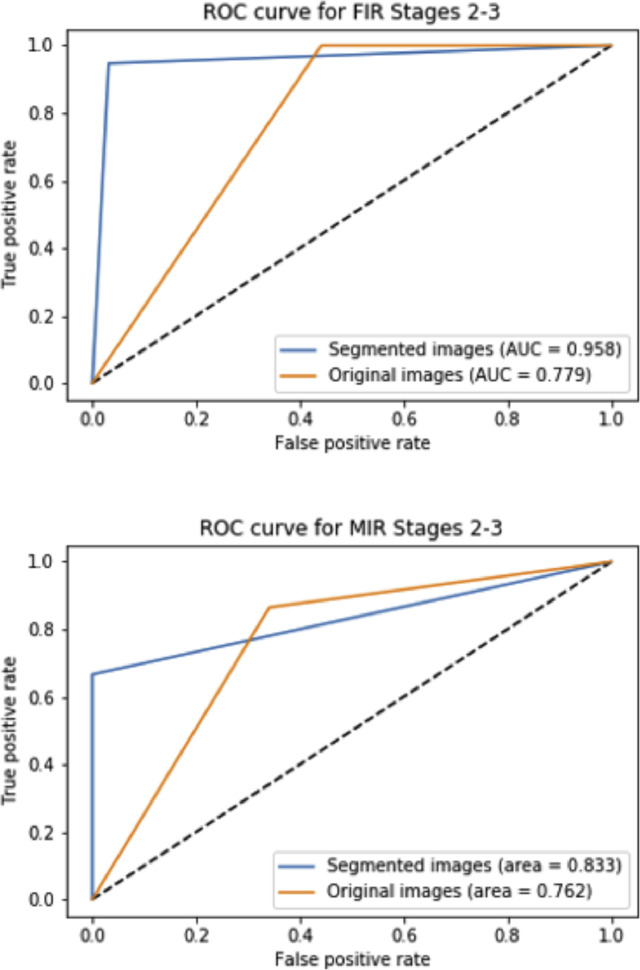
Prediction results comparison using original or segmented images for FIR (top) and MIR (bottom).

Fetal and Maternal Inflammatory Responses (FIR, MIR) are components of ascending infection and chorioamnionitis in pregnancy and predictive of infant sepsis [19]. To show the application potential of the MSL, we applied a pre-trained source-domain classification model to the target domain to predict if FIR and/or MIR of Stage 2 or 3 are observed within an image. The labels for training are from the collected pathological records (prepared via traditional histological exams), while the images in the target domain were annotated by a perinatal pathologist (from the images alone). Instead of using full translated images as the input, we fed the network with segmented images only occupied by cord and disk to remove distractions. We compare the ROC curves [5] of the FIR and MIR prediction either employing full images or segmented images in Fig. 6, where the AUC has been substantially enhanced when the segmented images are utilized for both cases. Specifically, the Area Under Curve(AUC) for FIR and MIR prediction changed from 0.78 and 0,76 to 0.96 and 0.83 correspondingly.

## 4 Conclusions

To enable the use of machine learning based pathology image analysis models in very different hospital environments, we have proposed a new unified pipeline adopting multi-region saliency-aware learning for cross-domain placenta image segmentation. Our approach guides the translation between domains by enforcing both the image-level and semantic-level consistency. By introducing the attention and saliency consistency constraints, the translation performance is substantially improved and the segmentation accuracy is enhanced. To our knowledge, this is the first approach for cross-domain placenta image segmentation, with real clinical datasets involving hundreds of patients, to demonstrate clinical relevance/viability in clinical practice. We showed successful use of our proposed model in instantly detecting significant pathological/abnormal indicators, MIR and FIR, which traditionally need histology to diagnose. This application lays the foundation for further pathological indicator analysis in real clinical situations.

## Declaration of Competing Interest

The authors declare that they have no known competing financial interests or personal relationships that could have appeared to influence the work reported in this paper.
